# Simplified sleep resistance test for daytime sleepiness detection

**DOI:** 10.5935/1984-0063.20200046

**Published:** 2021

**Authors:** Luis Darío Larrateguy, Carlos M. Pais, Luis I. Larrateguy, Santiago D. Larrateguy, Gaston Schlotthauer

**Affiliations:** 1 Centro Privado de Medicina Respiratoria, Sleep Medicine - Paraná - Entre Ríos - Argentina.; 2 Facultad de Ingeniería. Universidad Nacional de Entre Ríos, Carrera de Bioingeniería - Oro Verde - Entre Ríos - Argentina.; 3 Consejo Nacional de Investigaciones Cientíﬁcas y Técnicas, Laboratorio de Señales y Dinámicas no Lineales. Bioingeniería - Oro Verde - Entre Ríos - Argentina.

**Keywords:** Sleep Apnea Syndromes, Sleepiness, Wakefulness

## Abstract

**Objectives:**

Excessive daytime sleepiness (EDS) is a highly prevalent symptom that increases the risk of traffic accidents and deteriorates the quality of life. The diagnosis of EDS is difficult because of the complex infrastructure that is required. The new test here proposed assesses the ability of a simple test of simplify the detection of daytime sleepiness compared with the OSLER test.

**Material and Methods:**

In the new test, during 20 minute subjects were asked to pass a ﬁnger by a groove in response to a light emitting diode, inside dark glasses, which was lit for 1s in every three, with headphones that reduce the ambient noise and was compared with the OSLER test on each subject in random order.

**Results:**

The proposed method showed a sensitivity of 100% and a speciﬁcity of 61%, with a positive predictive value of 67% and negative predictive value of 100% when compared with the OSLER test. The value of area under the ROC curve was 0.81 (0.62-0.99), p=0.013. In a Bland-Altman plot, most of the latency times differences are in the 95% agreement interval (p=0.05). In addition, the conﬁdence interval of the mean and most of the positive results are above the zero line. The Cohens Kappa coefficient obtained is 0.58 (95% CI 0.29-0.88).

**Conclusion:**

In this sample of patients, the proposed method detects EDS in a similar way as OSLER test and can be performed in different environments without requiring special infrastructure or expert personnel.

## INTRODUCTION

Excessive daytime sleepiness (EDS) is a highly prevalent symptom (10 to 20% of the general population), that increases the risk of traffic accidents and deteriorates the quality of life^1^^,^^2^.

EDS is defined as the tendency to involuntarily fall asleep at inappropriate times and/or situations during hours when one should be awake^3^. While it can occur in healthy people during the premenstrual period, pregnancy or the postprandial period, it is considered pathological when it is associated with inadequate sleep, mainly sleep breathing disorders or schedule changes due to work rotating shifts, circadian rhythm disturbances, and alcohol and psychotropic drugs intake.

The excessive daytime sleepiness is one of the main symptoms of obstructive sleep apnea-hypopnea syndrome (OSAHS), and is related with severe public health consequences, such as traffic, domestic and at the work place accidents. For this reason, its assessment is important for physicians treating patient with OSAHS^1^^,^^2^.

This syndrome is highly prevalent in the general population and, for this reason, it should be a priority to detect and treat in symptomatic patients^3^. The diagnosis is made by overnight polysomnography (PSG) in a sleep laboratory. However, in cases with a high clinical suspicion of OSAHS, a simplified study as a respiratory polygraphy (RP) at home is enough. The existence of EDS, one of the most important symptoms in subjects suffering OSAHS, plus the report of snoring and apneas by bed partners, helps to evaluate the priority in conducting a simplified sleep study, decreasing in this way public health expenditures.

Moreover, the EDS as a symptom can be found in patients with other pathologies, such as restless leg syndrome, periodic limb movement disorder, insomnia, allergic rhinitis, gastroesophageal reflux disease, rheumatic diseases causing chronic pain, circadian rhythm sleep disorders in rotating shifts workers, jet lag, and other situations that cause poor sleep quality, increasing the importance of its diagnosis^1^.

EDS assessment is currently done subjectively with scales or self-administered questionnaires, as the Epworth Sleepiness Scale (ESS), Stanford Sleepiness Scale or Karolinska Sleepiness Scale^4^^,^^5^.

When it comes to objectively confirming EDS, neurophysiological test as the Multiple Sleep Latency Test (MSLT) and the Maintenance of Wakefulness Test (MWT) are used. The MSLT attempts to determine the time it takes to fall asleep in a favorable situation. On the other side, the MWT measures the ability to stay awake in the same situation, that is, resistance to sleep^2^^,^^5^^,^^6^.

Although it is preferable to use objective neurophysiological tests to confirm the presence of EDS instead of scales influenced by the subjectivity of the evaluated individual, these tests (MSLT and MWT) require complex infrastructure (sleep lab) and trained personnel, which increases the cost and makes difficult its use in the daily practice and in large populations^5^.

If the prevalence of EDS found in other populations is extrapolated, it would be between eight and twelve million people with sleepiness in Argentina^1^. The study of this population with current methods, such as MSLT and MWT, is very difficult; both because the associated costs and their lack of sufficient sleep labs and personnel with the skills required to perform these studies.

In 1997, Bennett et al.^7^, proposed the Oxford Sleep Resistance Test (OSLER), a simplified modification of the MWT, and a sleep resistance test that has been able to discriminate between patients with EDS and normal subjects.

In this test, the subject is seated in a dark room, isolated from ambient noise and is asked to press a springless button on a device hold in his hand, in response to a flash of light emitted by a diode that lights during a second every three seconds. The light source placed on a wall, two meters away. Four tests are performed at two-hour intervals and the sleep latency is calculated considering the onset of sleep when there is no response to seven consecutive pulses in 21 seconds (seven flashes of light). In case the subject does not fall asleep, the test stops after 40 minutes^7^. In its original proposal, the test was performed on 20 subjects (10 subjects with obstructive sleep apnea with EDS and 10 normal subjects without EDS and compared to MWT performed one day later. The OSLER was able to discriminate between normal and somnolent individuals. All normal subjects had a mean OSLER latency above 20 minutes and none of the patients with sleep apnea had latencies equal or greater than 20 minutes^7^, also verified in an OSLER validation study by Krieger et al.^8^.

However, Mazza et al.^9^, they found that the OSLER tests performed between 9:00 and 11:00 a.m. provide the same information in terms of percentage of patients classified as having EDS. Additionally, they found that the results of tests conducted at noon and over the next few hours, cannot distinguish between pathological patients from those with physiological sleepiness at these times. According to these data, it would not be necessary to perform the four tests of the original proposal and a single test between 09.00 and 11.00 a.m. would be sufficient^9^. Although it is a test more simpler and easier than MSLT and MWT, it needs trained staff, it takes time (four sessions of 40 minutes each), and a comfortable, quiet and dark room, which increases costs and hampers its realization in standard health institutions, limiting it to the scope of sleep laboratories.

To simplify the objective detection of daytime sleepiness, the OSLER test was modified to be portable, inexpensive, and easier to use.

The new sleep resistance test, called TRES (acronym in Spanish for **T***est de* **RE***sistencia al* **S***ueño*), does not need experienced staff, it takes place in a single 20-minute session between 9 a.m. and 11 a.m. and it can be done in any field without the need for special infrastructure. In this paper, TRES is compared with OSLER test.

## MATERIAL AND METHODS

### Subjects

Were recruited 23 patients who consulted for symptoms associated with sleep breathing disorders at department of pulmonary medicine, San Martín Hospital in Paraná, Entre Ríos, Argentina. This Hospital does not have the necessary infrastructure for performing sleep and EDS tests. For this reason, the patients were referred to the Clínica Modelo in Paraná.

The patients were informed about the characteristics of the study and an informed consent was signed by those who accepted to participate. The informed consent and the protocol were reviewed, evaluated, and approved by the Teaching and Research Committees of San Martín Hospital and Clínica Modelo.

### Protocol

The OSLER test, proposed by Bennett et al.^7^, in 1997, and validated by Priest et al.^10^ for the detection of EDS in 2001, was chosen as the reference test. The results of this test were compared prospectively with those obtained using the TRES test. All subjects underwent a full-night polysomnogram the night prior the EDS tests were performed.

The day after the PSG between 09:00 and 11:00 a.m., both the OSLER test and the TRES tests were performed, spaced five minutes apart. The order of the tests was randomly drawn for each patient. All tests were performed in the same room, which was adapted as described in similar studies^7^^,^^8^^-^^10^. Prior to the tests, patients were instructed to respond to the Spanish-validated version of the Epworth Sleepiness Scale^11^^,^^12^.

### Measurements

#### Nocturnal polysomnography

The night before the EDS tests, PSG was performed with an ATI Delphos^®^ device (Advantek SRL, Bs As, Argentina), in all patients for at least six hours, controlled by a polysomnographic technician.

#### OSLER

The OSLER test was performed by means of OSLER^®^device (Stowood Scientific Instruments Ltd., Common Road, Beckley, Oxford OX3 9UP, UK). The protocol described by Bennett et al.^7^ was applied, with some modifications as detailed here. In their original study, Bennett et al.^7^ proposed to perform four trials, separated by intervals of 2h. Each one of these sessions ended at the onset of sleep (which is defined as the lack of response to seven consecutive flashes) or, if sleep is not reached, at 40 minutes of the beginning of each trial.

According to the studies by Mazza et al.^9^, Krieger et al.^8^, and Priest et al.^10^, it was decided to perform a single trial with each test, between 09:00 and 11:00 a.m. with a maximum duration of 20 minutes if the subject did not give a response to seven consecutive light flashes in each study^8^^-^^10^.

#### TRES

**Figure 1:**
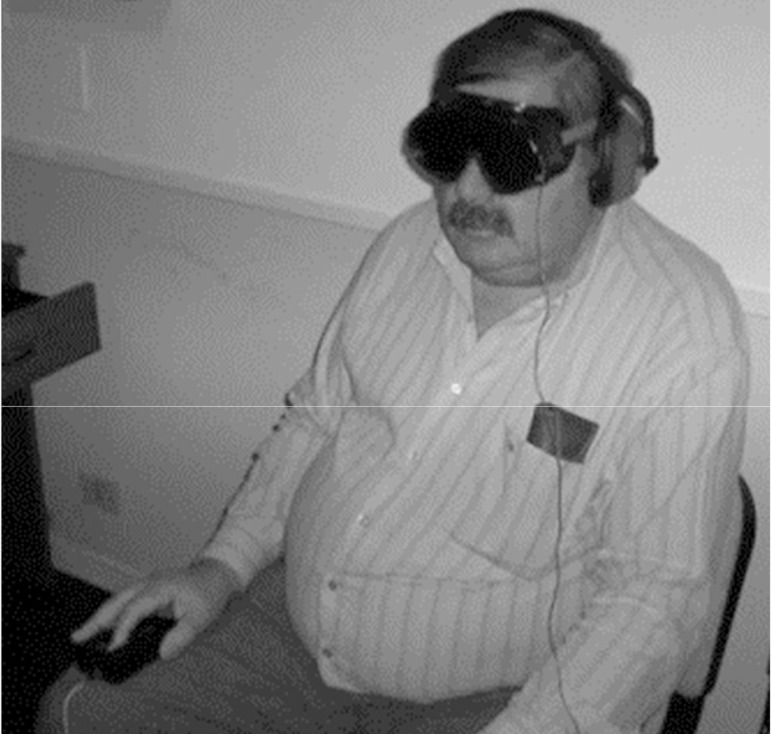
A patient performing the new test.

To perform the TRES test, each patient was sitting in a comfortable chair with dark glasses that did not allow the passage of light (Figure 1). Two LED (light emitting diode) were arranged inside the glasses, flashing during one second every three seconds. The influence of the external noise was reduced by headphones. Volunteers confirmed the view of the luminous flash using a device where they pass their index finger through a groove with an infrared beam. After seven consecutive flashes without a response, the TRES equipment beeps and the test ends. The time elapsed since the beginning until the end of the trial due to seven consecutive errors is interpreted as the sleep latency time. In accordance to previously described studies, in this work a 20 minutes single trial was performed, between 09:00 and 11:00 a.m., spaced five minutes apart from the OSLER test^7^^-^^10^.

## RESULTS

**Figure 2:**
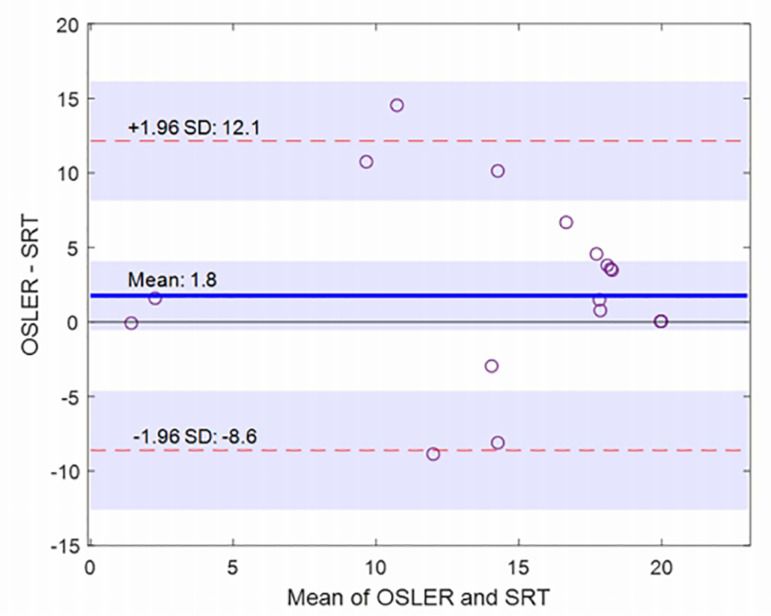
Bland and Altman plot for the sleep latency time by OSLER and TRES for seven errors.

In Table 1 we detail the information about each volunteer, and in Table 2 polysomnographic statistics are presented. The Figure 2 shows a Bland-Altman plot. It can be observed that the latency times for seven errors detection are within the 95% limits of agreement (*p*=0.05). Shaded areas represent the 95% confidence interval limits for mean and agreement limits. The zero line is within the confidence interval of the mean, which means that there is not a significant systematic difference between both tests.

**Table 1:** Data per patient: N: patient number. Rand: randomized first test. TRES: New Test. OSLER: OSLER test.

N	Rand.	Sex	Age	EPWORTH SLEEPINESS SCALE	HYPOPNEA INDEX APNEA	Latency in minutes for 7 errors with TRES	Latency in minutes for 7 errors with OSLER
1	TRES	F	52	14	9	15.57	12.57
2	OSLER	M	62	11	50	16.48	20.00
3	TRES	F	79	4	12	20.00	20.00
4	OSLER	M	63	15	23	16.57	20.00
5	OSLER	M	50	19	16	17.51	18.24
6	OSLER	F	47	9	29	20.00	20.00
7	OSLER	M	50	7	41	16.24	20.00
8	TRES	F	70	21	22	15.48	20.00
9	OSLER	M	41	17	94	16.48	7.57
10	TRES	F	55	16	11	1.51	1.39
11	OSLER	M	72	18	53	4.33	15.03
12	OSLER	M	52	19	32	1.51	3.06
13	OSLER	M	34	6	44	20.00	20.00
14	TRES	F	64	9	15	3.51	18.00
15	TRES	M	44	6	2	18.36	10.21
16	TRES	M	36	15	15	20.00	20.00
17	OSLER	M	41	14	35	20.00	20.00
18	TRES	F	36	16	0	20.00	20.00
19	OSLER	F	34	11	1	9.24	19.33
20	OSLER	M	82	12	10	13.36	20.00
21	OSLER	M	52	10	58	17.12	18.57
22	TRES	M	60	14	75	20.00	20.00
23	TRES	M	47	15	6	20.00	20.00

**Table 2:** Polysomnographic statistics.

Polysomnographic Parameters	MEDIAN Interquartil Range	Normal Values
Total recording time (minutes) TRT	406.40 (27.72)	360-480
Total sleep time (minutes) TST	376.46 (44.05)	324-422
Sleep latency (minutes)	11.71 (21.59)	>15
Sleep efficiency (percentage)	92.20 (8.68)	>85 %
Time in No REM (minutes) [% of TST]	309.68 (99.30) [82.26]	80% of TST
Time in REM (minutes) [% of TST]	43.58 (71.22) [11.50]	20% of TST

Considering the Sleep Resistance Test as a diagnostic test for detecting seven consecutive errors, and taking the OSLER test as the reference, the TRES sensitivity was 100% and the specificity was 61% with a positive predictive value of 67% and negative of 100%. The area under the ROC curve was 0.81 with a 95% confidence interval of (0.62-0.99), being statistically significant (*p*=0.013).

The Cohen’s kappa coefficient was 0.58 with a 95% confidence interval of (0.29-0.88) for seven errors.

## DISCUSSION

This paper presents a new test and a device that objectively detects EDS in a simple way, which can be used in any field by non-expert personnel without special infrastructure. A device with these characteristics could allow the detection of those patients who should be studied promptly to diagnose and correct the cause of excessive daytime sleepiness and thus save health resources^13^.

The proposed TRES test consists in a single trial between 9:00 a.m. and 11:00 a.m., with a maximum duration of 20 minutes until seven consecutive errors are committed. It was compared with OSLER test, which proved to be an objective diagnostic method of EDS^7^^-^^10^, in a sample of patients with poor sleep quality, with little REM sleep, short sleep latency and high sleep efficiency (Table 2). Over 70% of patients had a result greater than 10 in the Epworth scale. According to these data, it can be presumed that in the studied population, EDS should be confirmed by objective methods.

Analyzing the Bland and Altman plot, the differences in latency times for the detection of seven errors in both methods are not clinically significant. Most of data fall within the 95% agreement limits (*p*=0,05), with only two of them remaining just outside these limits. However, it can be seen in Figure 2 that these two data are within the confidence intervals for the estimates of the agreement limits, indicated by the shading^14^^,^^15^; in this Figure it can also be noticed that the zero line is within the confidence interval of the mean, which indicates that the bias is not significant. In addition, most of the results are above the zero line, indicating that the TRES method would detect seven errors earlier than the OSLER.

Considering OSLER as the reference test, the sensitivity of TRES is 100%, while specificity, positive predictive value and negative predictive value are 61%, 67%, and 100%, respectively. The area under the ROC curve for TRES reaches a value of 0.81, with a 95% confidence interval of (0.62-0.99), being statistically significant (*p*=0.013).

How can be these results clinically interpreted? Are the measurements obtained by both method similar? Does the TRES method detect seven errors before the OSLER, or are there false-positives?

In a study by Priest et al.^10^, the authors reported false-positive results in two of the ten subjects undergoing OSLER test. These two volunteers committed all seven errors despite being awake, as it was clinically observed^10^. The authors of that study assume that these false positives can occur because of lack of attention, frequent blinking, or decreased wakefulness of the patients. Another possible reason of a false positive is that the volunteers could have pressed the button in response to the flashes of light too slightly, at undetected levels by the OSLER system^10^.

On the other hand, the standard way of analyzing the OSLER test does not detect abnormal fluctuations in the level of wakefulness during the test that could give rise to false negatives. In this work, we were observing the volunteers behavior through a video system during the OSLER trials. We detected fluctuations in the level of wakefulness in several patients during the OSLER test: they tried to be alert using different techniques, as looking sideways, hitting his face, standing and sitting on the chair, or opening and closing his eyes.

These alterations in the level of wakefulness of patients while performing OSLER were not observed while performing the TRES.

In summary, in this sample of patients, both methods showed similar results to detect EDS, but the method proposed here, TRES, detects EDS in a shorter time than with the OSLER method, and can be performed in any area without requiring infrastructure special or expert staff.

In future studies, it could analyze the error profile and discriminate patterns of possible subjects with excessive daytime sleepiness.

On the other hand, when it comes to evaluating sleepiness in children, developing and evaluating tools, takes a lot of time and work^4^^,^^16^. And while MSLT and MWT are the best objective measures currently available to characterize the ability to fall asleep and the ability to stay awake, even in children, they are also not perfect tests. MSLT should not be used as a screening tool. Because the MSLT and MWT are in-laboratory tests, it is important develop novel techniques that provide reliable assessment of sleepiness and wakefulness in the real work environment over extended periods^16^^-^^19^.

Thus, in the real world, TRES could be an objective screening tool for excessive daytime sleepiness easy to use in different age groups in future studies.
